# *CFTR* Gene Mutations in the Egyptian Population: Current and Future Insights for Genetic Screening Strategy

**DOI:** 10.3389/fgene.2017.00037

**Published:** 2017-03-31

**Authors:** Ayman S. El-Seedy, Hanaa Shafiek, Alain Kitzis, Véronique Ladevèze

**Affiliations:** ^1^Department of Genetics, Alexandria UniversityAlexandria, Egypt; ^2^EA3808, Groupe Génétique des Maladies Rares, Université de PoitiersPoitiers, France; ^3^Department of Chest Diseases, Alexandria UniversityAlexandria, Egypt; ^4^Centre Hospitalier Universitaire de PoitiersPoitiers, France

**Keywords:** Cystic fibrosis, *CFTR* mutations, genetic screening, Egyptian patients

Cystic Fibrosis (CF) is the most common lethal rare genetic disease in the Caucasian populations. It is caused by a variety of sequence alterations in the Cystic Fibrosis Transmembrane Regulator (*CFTR*) gene. In Caucasian, one over 3,500 new born children suffers from the disease and one over 30 of them is at least carrier of a severe mutation in the *CFTR* gene. CF and *CFTR*-related disorders (*CFTR-*RDs) are two distinct clinical outcomes of the gene mutations. The CF mutation induces a severe phenotype involving different organs, whereas a *CFTR-*RD mutation induces less life-threatening symptoms with three main clinical entities including congenital bilateral absence of the vas deferens (CBAVD), acute recurrent or chronic pancreatitis, and disseminated bronchiectasis (Bombieri et al., 2011).

In the Arab countries, the spectrum of CF mutations, incidence and prevalence of the disease are largely unknown in the Arab populations (Wei et al., 2006). This is due to the lack of disease awareness, and diagnosis facilities that mislead the identification of CF during many decades. Additionally, epidemiological studies that realized were revealed a distinguished mutational spectrum between Arab countries if compared to White-European populations. Furthermore, Arab Mediterranean countries have a different *CFTR* mutational profile if compared to the Arabian Peninsula.

Egypt as Mediterranean North African country, this strategic position attracted many invaders throughout its history. Therefore, in addition to its Pharaonic origin, gene flow to its population occurred from the Ethiopian, Greco-Roman, Arab, Turkish, French and English settlers (Temtamy et al., 2010). The common heritage among the countries bordering the Mediterranean is not restricted to historical or cultural aspects. There are considerable commonalities in the gene pools of the Mediterranean Northern and Southern countries. This “genetic sharing” has resulted from considerable human movements (i.e., migration, invasion, and trade) throughout history in this area (Temtamy et al., 2010). Consequently, Egypt is not like other Arab countries and mutations in the *CFTR* gene have been influenced by gene flow coming from different populations. Furthermore, the high rate of consanguinity, infant and neonatal mortality in the Egyptian society will, therefore, increase CF incidence and private mutations. Indeed, another important issue is the increase number of *CFTR*-related disorders patients such as idiopathic bronchiectasis, congenital bilateral absence of the vas deferens, idiopathic (non-alcoholic) pancreatitis, and severe sinusitis in the Egyptian population. This observation is in agreement with data previously published (Lissens et al., 1999; Hussein et al., 2011; Fathy et al., 2016).

In Egypt, there is no available data on the nature and frequency of CF gene mutations. There are a few previous reports of *CFTR* gene mutations in Egyptian patients that have been published. In these papers, a CF screening strategy of *CFTR* mutations was realized using commercial kits or a panel defined by the American College of Medical Genetics and Genomics (ACMG) and American College of Obstetricians and Gynecologists (ACOG), (Naguib et al., 2007; El-Falaki et al., 2014; Fathy et al., 2016; Shahin et al., 2016), which is not suitable for screening of the *CFTR* gene mutations in the Egyptian population. This emphasizes the need for establishing a correct genetic screening for CF mutations in Egyptian patients and to determine the carrier status in their healthy relatives.

In our recent study, we have performed a complete *CFTR* gene screening in CF or *CFTR-RD* patients from Alexandria, Northern Egypt, by direct sequencing of the entire CFTR gene that identified four novel CF mutations in the *CFTR* gene amongst 13 other known mutations in the other populations (Figure 1A). Furthermore, this is the first comprehensive profile of *CFTR* gene mutations and their corresponding haplotypes in the Egyptian population (El-Seedy et al., 2016). Besides, *CFTR* mutations in the other Egyptian regions have not been analyzed. Thus, the identification of *CFTR* mutations is become increasingly important in genetic counseling and prenatal diagnosis particularly in families with multiple affected children (Wang et al., 2000).

**Figure 1:**
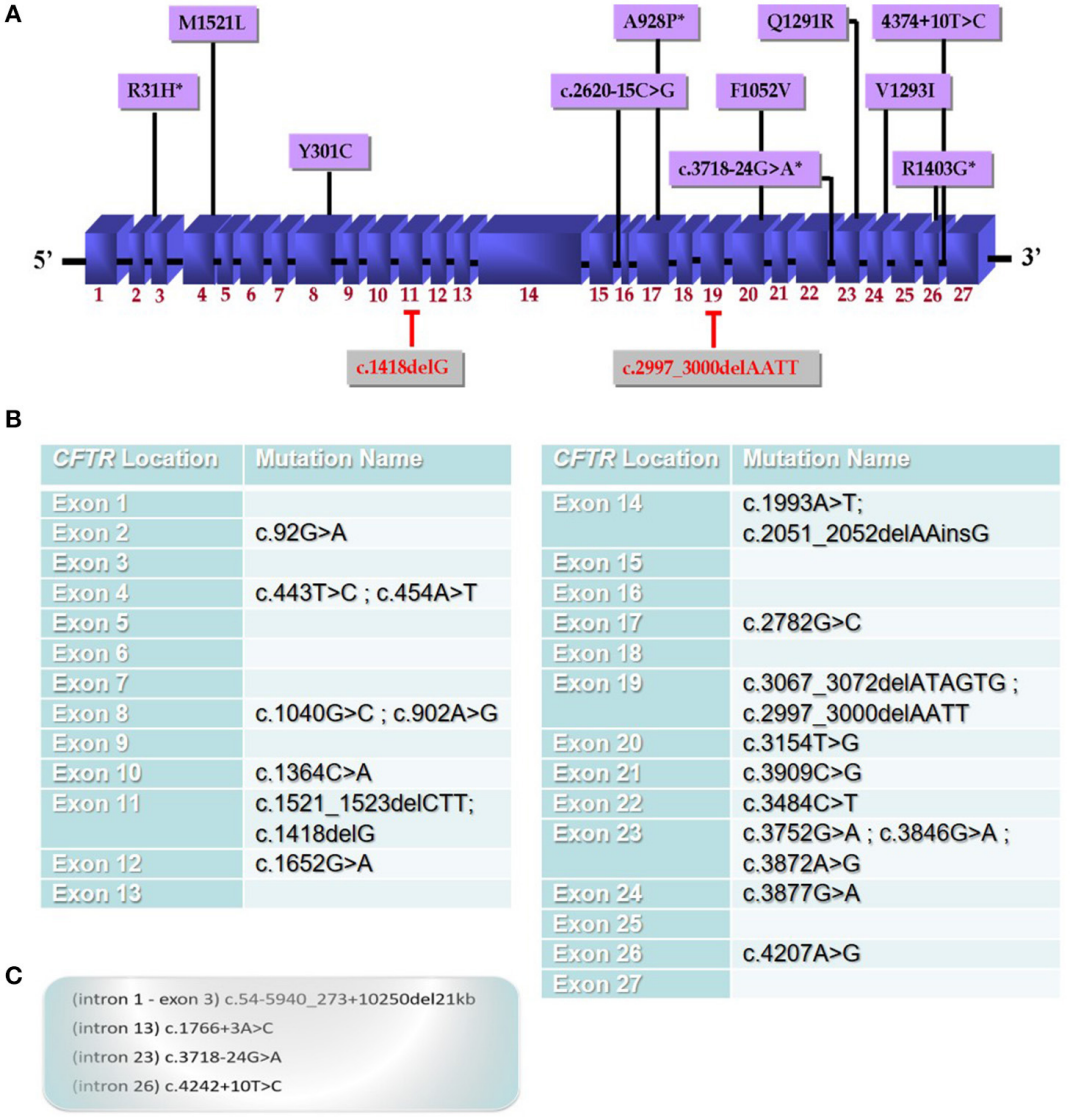
**Mutational spectrum of *CFTR* gene mutations in the Egyptian population. (A)** *CFTR* mutations identified in our recent investigation of Egyptian CF or *CFTR-*RD patients from Alexandria city, Northern Egypt which performed a complete *CFTR* gene screening by direct sequencing of the entire *CFTR* gene. This study is the first to identify novel mutations (indicated with a star^*^) in the Egyptian population amongst 13 other known mutations that appear to be a specific to Egyptian CF patients (2); **(B)** a panel of the most common mutations detected in the Egyptian population; **(C)** intronic mutations identified in the Egyptian patients that will be also present in the panel.

According to available data of *CFTR* gene mutations, we propose general national screening panel comprising 27 mutations reported in Egyptian patients until now. This will help to detect about 50% of *CFTR* gene mutations in Egypt and further extensive studies will help to improve this panel. The establishment of the first panel of the *CFTR* gene mutations in the Egyptian population (Figures 1B,C) will help physicians for designing an appropriate strategy for future genetic diagnosis for patients and families at risk.

In this regard, defining a population-specific mutational panel for CF in Egyptian population including: c.92G>A (exon 2), c.454A>T (exon 4), c.902A>G (exon 8), c.1418delG (exon 11), c.2620-15C>G (intron 15), c.2782G>C (exon 17), c.2997_3000delAATT (exon 19), c.3154T>G (exon 20), c.3718-24G>A (intron 23), c.3872A>G (exon 23), c.3877G>A (exon 24), c.4207A>G (exon 26), c.4242+10T>C (intron 26) mutations identified in our recent study (Figure 1A), and together with previously reported mutations in Egyptian patients (Figures 1B,C) [c.1766+3A>C (intron 13), c.1993A>T (exon 14), c.3909C>G (exon21), c.1521_1523delCTT (exon11), (Naguib et al., 2007); c.54-5940_273+10250del21kb (intron 1-exon 3), c.443T>C (exon 4), c.1040G>C (exon8), c.1364C>A (exon10), c.1652G>A (exon12), c.2051_2052delAAinsG (exon 14), c.3067_3072delATAGTG (exon19), c.3484C>T (exon 22), c.3846G>A (exon 23), (Shahin et al., 2016); c.1040G>C (exon 8), c.1364C>A (exon 10), c.3484C>T (exon 22), c.3752G>A (exon 23), c.3846G>A (exon 23), (Fathy et al., 2016)] should be highly recommended for Egyptian patients. However, full clinical manifestations of additional CF and *CFTR-*RDs patients could lead to clarify the pathogenicity of these mutants in this population.

The overall purpose of this paper, in our opinion, is to develop and validate a strategy for increasing the sensitivity of *CFTR* screening in Egyptian population. This strategy includes establishing of an Egyptian CF Registry Network (ECFRN) where there is no reliable estimates on the number of CF patients and clinical pattern as well as the most common *CFTR* mutations in this population. Hence, this registry is essential in order to have data about prevalence and incidence of CF in Egypt for improving clinic diagnostic outcomes and planning health policies. In addition, an extensive molecular analysis of *CFTR* gene using next-generation sequencing (NGS) technology combined with multiplex ligation-dependent probe amplification (MLPA) will provide a wider coverage of the *CFTR* locus and expand our proposed panel in CF molecular diagnosis.

In summary, our findings confirm the importance of direct gene sequencing of the entire *CFTR* gene in combination with molecular and functional studies (in isolation and in complex alleles) in order to perform a correct genetic counseling and to estimate the prevalence of the carriers in the general population. Moreover, collaborative studies, on the clinical and fundamental levels, are necessary to give an adequate genotype-phenotype correlation. Thus, sharing the obtained data between *CFTR* laboratories, clinical research centers and concerned communities in Egypt are also from a great importance to ensure a progression in mutation researches and to help clinicians in giving a suitable prenatal diagnosis, patients advising and treatments prescription when available.

## Author contributions

AE: Wrote the paper. HS, AK, and VL: Revised the paper.

### Conflict of interest statement

The authors declare that the research was conducted in the absence of any commercial or financial relationships that could be construed as a potential conflict of interest.
